# Construction of Eukaryotic Expression Vector with *mBD1-mBD3* Fusion Genes and Exploring Its Activity against Influenza A Virus

**DOI:** 10.3390/v6031237

**Published:** 2014-03-13

**Authors:** Wanyi Li, Yan Feng, Yu Kuang, Wei Zeng, Yuan Yang, Hong Li, Zhonghua Jiang, Mingyuan Li

**Affiliations:** 1 Department of Microbiology, West China School of Preclinical and Forensic Medicine, Sichuan University, Chengdu 610041, China; E-Mails: hxliwanyi@126.com (W.L.); yanfeng_1983@126.com (Y.F.); kuangyu20001113@163.com (Y.K.); h_zwcn@163.com (W.Z.); yangyuan1114@163.com (Y.Y.); jjzz1879@163.com (Z.J.); 2 West China Second University Hospital, Sichuan University, Chengdu 610041, China; E-Mail: lihong09@163.com; 3 State Key Laboratory of Oral Diseases, Sichuan University, Chengdu 610041, China

**Keywords:** influenza A virus, mouse beta-defensin, pcDNA3.1(+)/*mBD1-mBD3*, overlap-PCR, anti-virus activity

## Abstract

Influenza (flu) pandemics have exhibited a great threat to human health throughout history. With the emergence of drug-resistant strains of influenza A virus (IAV), it is necessary to look for new agents for treatment and transmission prevention of the flu. Defensins are small (2–6 kDa) cationic peptides known for their broad-spectrum antimicrobial activity. Beta-defensins (β-defensins) are mainly produced by barrier epithelial cells and play an important role in attacking microbe invasion by epithelium. In this study, we focused on the anti-influenza A virus activity of mouse β-defensin 1 (mBD1) and β defensin-3 (mBD3) by synthesizing their fusion peptide with standard recombinant methods. The eukaryotic expression vectors pcDNA3.1(+)/*mBD1-mBD3* were constructed successfully by overlap-PCR and transfected into Madin-Darby canine kidney (MDCK) cells. The MDCK cells transfected by pcDNA3.1(+)/*mBD1-mBD3* were obtained by G_418_ screening, and the *mBD1-mBD3* stable expression pattern was confirmed in MDCK cells by RT-PCR and immunofluorescence assay. The acquired stable transfected MDCK cells were infected with IAV (A/PR/8/34, H1N1, 0.1 MOI) subsequently and the virus titers in cell culture supernatants were analyzed by TCID_50_ 72 h later. The TCID_50_ titer of the experimental group was clearly lower than that of the control group (*p* < 0.001). Furthermore, BALB/C mice were injected with liposome-encapsulated pcDNA3.1(+)/*mBD1-mBD3* through muscle and then challenged with the A/PR/8/34 virus. Results showed the survival rate of 100% and lung index inhibitory rate of 32.6% in pcDNA3.1(+)/*mBD1-mBD3*group; the TCID_50_ titer of lung homogenates was clearly lower than that of the control group (*p* < 0.001). This study demonstrates that mBD1-mBD3 expressed by the recombinant plasmid pcDNA3.1(+)/*mBD1-mBD3* could inhibit influenza A virus replication both *in vitro* and *in vivo*. These observations suggested that the recombinant mBD1-mBD3 might be developed into an agent for influenza prevention and treatment.

## 1. Introduction

Influenza A virus (IAV) is a major cause of life-threatening respiratory tract diseases worldwide. A human influenza (flu) pandemic could cause 20% of the global population to become ill. In past decades, avian flu H5N1 has emerged among chickens and human in some developing countries, so that close to 30 million people would need to be hospitalized, a quarter of whom would die within a few months [1]. As is well known, in April 2009, tens of thousands of people were infected and more than one hundred died in the new H1N1 influenza (Human Swine Flu) global pandemic within only one month [2]. In March 2013, avian flu H7N9 subtype emerged in China, and hundreds people have been infected. Today, although annual vaccination is the primary strategy for preventing infections, antiviral drugs play an important role in the control of influenza [3,4]. However, some influenza viruses, even including the H5N1, have demonstrated resistance to antiviral drugs such as adamantine derivatives and neuraminidase inhibitors [5,6]. The increasing appearance of resistant strains of influenza virus highlights our need to search for alternative treatment strategies. 

The innate immune system is the first line of defense against invading pathogens, and defensins are the key elements of this system. Defensins are well known for their broad-spectrum antimicrobial activity, including against bacteria, fungi, and viruses [7,8]. Structurally, defensins are small (2–6 kDa) cationic, cysteine-rich peptides. Based on the spatial distribution of their six-cysteine residues and the connectivity of the disulfide bonds, defensins can be classified into three categories: α-, β-, and θ-defensins [9]. The β-defensins are predominantly produced by barrier epithelial cells and play an important role in blocking microbial invasion by way of the epithelium. Additionally, β-defensins can modulate the host’s cell-mediated immunity via cytokine expression, providing an interface between innate and adaptive immune response [10]. Due to their unique mechanism of action, β-defensins are expected to be the ideal therapeutic agents mitigating the problem of acquired drug resistance [11].

Both human β-defensins and mouse β-defensins (mBD) all have been shown to be particularly significant in airway and lung host defense [12,13]. One study shows that the *mBD* gene is a length of conservative nucleotide sequence which has higher homology with the human β-defensins gene [14]. Mouse β-defensin1 (mBD1) and β-defensin3 (mBD3) have been confirmed to have definite antimicrobial activity [15]. However, information on the combination antimicrobial activity of mBD1 and mBD3 has been scarce over the past decade. Especially the combination effects of mBD1 and mBD2 on influenza virus have been poorly studied. Therefore, we focused on the anti-IAV activity of mBD1 and mBD3 by synthesizing their fusion peptide with standard recombinant methods in this study. A eukaryotic expression vector pcDNA3.1(+)/*mBD1-mBD3*, was constructed and its potential to inhibit IAV in MDCK cells and mice were investigated.

## 2. Experimental Section

### 2.1. mBD1-mBD3 Fusion Genes Amplification and Synthesis

The cDNA for mature mBD1 and mBD3 were amplified using polymerase chain reaction (PCR) from the pcDNA3.1(+)*-mBD1* plasmid and pcDNA3.1(+)*-mBD3* plasmid, which have been constructed in our laboratory by Dr. Yue-ling Wang and Dr. Yan Jiang (Sichuan University, Chengdu, China), respectively. The forward and reverse primers (Table 1) specific to *mBD1* and *mBD3* were designed according to the coding sequence of mBD1 and mBD3 (GenBank Accession No. AA065510 and AF092929) and synthesized by Invitrogen (Shanghai, China). Forward primer for *mBD1* (*mBD1*-F) and reverse primer for *mBD3* (*mBD3*-R) contained a restriction site for *Eco*R I and *Xho* I (underlined), respectively, and reverse primer for *mBD1* (*mBD1*-R) owned an overlapping sequence of 15 bp (bold) with forward primer for *mBD3* (*mBD3*-F). The conditions of PCR amplification were as follows: 94 °C for 4 min; 30 cycles of 94 °C for 30 s; 54 °C for 35 s; 72 °C for 30 s; and 72 °C for 10 min. PCR products were separated and purified by agarose gel electrophoresis. Then, *mBD1* and *mBD3* genes were connected with a polypeptide linker Gly_4_Ser by overlap-PCR for constructing the chain of *mBD1-mBD3* fusion genes. The purified *mBD1* and *mBD3* PCR products were used as a template in overlap-PCR with *mBD1*-F and *mBD3*-R as primers and the following conditions: 94 °C for 4 min; 30 cycles of 94 °C for 30 s, 56 °C for 35 s, 72 °C for 30 s; and 72 °C for 10 min. The overlap-PCR product was separated and identified by agarose gel electrophoresis.

**Table 1:** Primers used for the amplification of *mBD-1* and *mBD-3*.

Template	Forward primer 5'-3'	Reverse primer 5'-3'	Size of product
*pcDNA3.1(+)-mBD1* plasmid	5'-GCGGAATTCATGAAAACTCATTACTTTCTCCTGG-3'	5'-**AGACCCGCCTCCACC**GCTCTTACAACA-3'	216 bp
*pcDNA3.1(+)-mBD3* plasmid	5'-**GGTGGAGGCGGGTCT**AAAATCAACAAT-3'	5'-GCCTCGAGCTATTTTCTCTTGCAGCATTTG-3'	209 bp

### 2.2. The Construction of Recombinant Plasmid pcDNA3.1(+)/mBD1-mBD3

The overlap-PCR product was cleaved by *Eco*R I and *Xho* I (Fermentas Co. Ltd, Shenzhen, China), and the *mBD1-mBD3* fragment was inserted into similarly digested pcDNA3.1(+) (Invitrogen, Shanghai, China) vector with T4 DNA ligase (Fermentas Co. Ltd, Shenzhen, China) at 16 °C to construct the expression plasmid named pcDNA3.1(+)*/mBD1-mBD3*. The inserted sequences were confirmed by PCR, restriction enzyme digestion analysis with *Eco*R I and *Xho* I and sequencing using the ABI 377 DNA Analyzer (Invitrogen, Shanghai, China).

### 2.3. Screening of Stable Expression MDCK Stains Transfected with pcDNA3.1(+)/*mBD1-mBD3*

The final concentration of MDCK cells were adjusted to 2 × 10^5^–5 × 10^5^/mL with DMEM (Gibco, Grand Island, NY, USA) supplemented with 10% bovine serum, 100 U/mL penicillin and 100 µg/mL streptomycin. MDCK cells were transfected by pcDNA3.1(+)/*mBD1-mBD3* and pcDNA3.1(+), respectively, using DNA-penetrator Transfection Reagent (Ribo Biotech Co. Ltd, Guangzhou, China) according to the instructions supplied by the manufacturer. Each plasmid was repeated three times through three wells and PBS was used as a mock transfected control. 48 h after transfection, stable screen was carried out using 600 µg/mL G_418_ for 20 days to obtain MDCK with a stable content pcDNA3.1(+)/*mBD1-mBD3*, named MDCK-pcDNA 3.1(+)/*mBD1-mBD3*, and MDCK with a stable content pcDNA3.1(+), named MDCK-pcDNA 3.1(+).

### 2.4. RT-PCR Analysis of *mBD1-mBD3* Fusion Gene Expression in MDCK

Total RNA was isolated from MDCK-pcDNA 3.1(+)/*mBD1-mBD3* and MDCK-pcDNA 3.1(+) with TRIzol (Invitrogen, Shanghai, China) according to the instructions provided by the manufacturer and stored at −70 °C. Reverse transcription of RNA was carried out using the First Strand cDNA Synthesis Kit (Fermentas, Hanover, MD) with *mBD1*-F and *mBD3*-R as primers and the following conditions: 94 °C for 4 min; 30 cycles of 94 °C for 30 s; 54 °C for 35 s; 72 °C for 30 s; and 72 °C for 10 min. Expression of *mBD1-mBD3* was quantified with reference to that of the MDCK β*-actin* (106 bp) gene (forward primer: 5'-GTCCCTGCCATGTATGTCGC-3'; reverse primer: 5'-ACATTGTGGGTGACACCGTC-3'). RT-PCR product was identified by agarose gel electrophoresis.

### 2.5. Indirect Immunofluorescence Analysis of *mBD1-mBD3* Fusion Protein Expression in MDCK

MDCK-pcDNA 3.1(+)/*mBD1-mBD3* and MDCK-pcDNA 3.1(+) were cultured in 6-well plates for 24 h. Cells were washed with PBS for three times, then fixed for 30 min with 0.4% paraformaldehyde (2 mL/well) , followed by a 30 min incubation in 0.5% Triton100 (2 mL/well). Washing again, rabbit serum (Fermentas Co. Ltd, Shenzhen, China) was used to block the blank, and then the cells were incubated overnight at 4 °C with goat anti-mouse mBD1 antibody (Abnova Co., Taibei, Taiwan) and rabbit anti-mouse mBD3 antibody (Santa Cruz Biotechnology, Inc., Santa Cruz, CA, USA) diluted 1:500 in PBS separately, followed by a 30 min incubation at room temperature with rabbit anti-goat IgG-RBITC (Fermentas, Hanover, MD, USA) and goat anti-rabbit IgG-FITC (Fermentas, Hanover, MD, USA) diluted 1:50 separately. Samples were washed three times in PBS after each incubation step. The expression of mBD1-mBD3 fusion proteins was reflected by fluorescence under fluorescent microscope.

### 2.6. Inhibition of IAV Replication in MDCK-pcDNA 3.1(+)/*mBD1-mBD3*

MDCK-pcDNA 3.1(+)/*mBD1-mBD3*, MDCK-pcDNA 3.1(+)/*mBD1*, MDCK-pcDNA 3.1(+) and MDCK were digested and adjusted to a final concentration of 1 × 10^5^/mL before the cells were cultured. When the cells had grown to 90% confluence in 96-well plates, cell culture media were removed and cells were washed two times in PBS to remove bovine serum. Different IAV (strain A/PR/8/34 H1N1, 0.1 MOI) dilution, from 10^−1^ to 10^−8^ (100 μL/well) were dropped into 3 wells for each dilution slightly and incubated for 1 h at 37 °C in a 5% CO_2_ incubator. IAV were removed and cells were washed three times in PBS, before cells were incubated with DMEM supplemented with 10% bovine serum (200 μL/well) and observed for cytopathic effect (CPE) under light microscopy. The 50% tissue culture infectious dose (TCID_50_) was obtained through calculating by the Reed-Muench method and −log10 of each TCID_50_ was analyzed with SPSS v11.5 software [16].

### 2.7. Immunohistochemistry Analysis of *mBD1-mBD3* Gene Expression in Vivo

Recombinant plasmid pcDNA3.1(+)/*mBD1-mBD3*, pcDNA3.1(+)/*mBD1* and pcDNA3.1(+)/*mBD3* were purified (large scale) by alkaline lysis and phenol-chloroform extraction. Positive ionic lipofectin (Invitrogen, Shanghai, China) encapsulated plasmids DNA were prepared by the Department of Pharmacy (West China School of Pharmacy, Sichuan University, Chengdu, China). The final ratio of positive ionic lipofectin and DNA is 1 nmol/μg. Forty-eight female BALB/C mouse (Laboratory Animal Center of Sichuan University, Chengdu, China) were divided into four groups: pcDNA3.1(+)/*mBD1-mBD3*, pcDNA3.1(+)/*mBD1*, pcDNA3.1(+)/*mBD3* and pcDNA3.1(+) negative control. In order to benefit from the diffusion of plasmid DNA, all mice were first inoculated with 50 μL of 25% cane sugar solution by intramuscular injection at the right quadricep femoris followed by inoculation with 50 μL of positive ionic lipofectin encapsulated recombinant plasmid DNA and pcDNA3.1(+) plasmid DNA at the same site according to the groups defined above. Two mice were inoculated with PBS at the same site as normal control. After inoculation, at 4 h, 8 h, 12 h, 24 h, 36 h and 72 h, the inoculated muscle tissues were excised from two euthanized mice and fixed in 4% paraformaldehyde overnight. The PBS control mice underwent the same treatment at 24 h. After being dehydrated by a series of ethanol and cleared in xylene, the fixed muscle samples were prepared into 5 µm sections. The sections were blocked for 30 min with 5% goat serum. The appropriate diluted (1:100) goat anti-mouse mBD1 antibody was added and incubated at 4 °C overnight. After washing, the rabbit anti-goat IgG labeled with biotin (Boster, Wuhan, China) was applied and incubated for 40 min at 37 °C. After samples were washed in PBS, Strept Avidin-Biotin Complex (SABC) was added and incubated for 30 min at 37 °C. Then coloration was proceeded in 3,3-Diaminobenzidine (DAB) substrate. Finally, the reactions were stopped with PBS and finalized with neutral balsam. The results were checked with light microscope.

### 2.8. Inoculation with Plasmids DNA in BALB/c Mice

Sixty female BALB/c mice (Laboratory Animal Center of Sichuan University, Chengdu, China) aged between four and six weeks were divided into five groups: pcDNA3.1(+)/*mBD1-mBD3*, pcDNA3.1(+)/*mBD1*, pcDNA3.1(+)/*mBD3*, pcDNA3.1(+) and PBS. pcDNA3.1(+) and PBS were designed as negative control and normal control.

Mice were inoculated with positive ionic lipofectin encapsulated plasmid DNA and PBS according to the groups defined above by intramuscular injection. Methods and dosage of injection were similar to what was previously described. Mice were anesthetized and challenged 36 h post-inoculation with 50 μL of 10× LD_50_ IAV (strain A/PR/8/34 H1N1) administered via intra-nasal inoculation.

### 2.9. Lung Index and Lung Index Inhibitory Rate Calculation

Three days after the challenge, three mice were chosen at random from each group and euthanized. After being weighed, the euthanized mice were dissected aseptically and lungs were excised. Lungs were weighed and lung index ((weight of lung/weight of mouse) × 100%) and lung index inhibitory rate ((average lung index of the negative control group − average lung index of the experimental group)/average lung index of the negative control group) × 100%) were calculated. Data were analyzed using a one-way ANOVA statistical method. A value of *p* < 0.05 was considered significant.

### 2.10. Influenza Virus Titrations for Lung Homogenates

Homogenates of the right lung tissue were prepared and centrifuged at 1500 rpm for 10 min. The supernatant was collected to be lung suspension. After MDCK cells were digested and grown to monolayer in 24-well plates, cell culture media were removed and cells were washed two times in PBS to remove bovine serum. Different lung suspension dilution, from 10^−1^ to 10^−8^ were dropped into 3 wells for each dilution slightly and incubated for 48 h at 37 °C in 5% CO_2_ incubator. CPE was observed under light microscopy. TCID_50_ titer was obtained through calculating by Reed-Muench method and −log10 of each TCID_50_ titer was analyzed with SPSS v11.5 software [16].

### 2.11. Pathological Changes in the Lung

The middle lobe of the left lung from each mouse was fixed with 10% neutral formaldehyde solution for 16 to18 h. Tissues were embedded in wax and cut into 5 µm slices for hematoxylin and eosin (H and E) staining, and pathological changes were observed and recorded through light microscopy by the Department of Pathology (West China Hospital, Sichuan University, Chengdu, China).

### 2.12. The Mice Survival Rate after IAV Challenge

Following the IAV challenge, the experimental BALB/c mice were observed and weighed regularly for 20 days. The rate of survival was defined as the percentage of surviving mice and data were analyzed using a χ^2^ test. A value of *p* < 0.05 was considered significant.

## 3. Results

### 3.1. PCR Amplification and Restriction Enzyme Digestion Analysis of pcDNA3.1(+)/*mBD1-mBD3*

**Figure 1:**
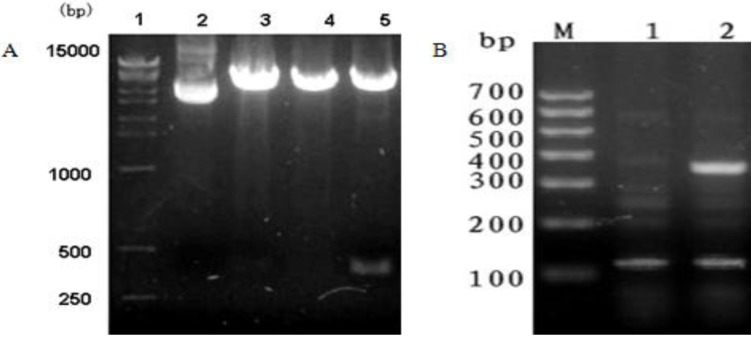
Restriction enzyme digestion analysis of pcDNA3.1(+)/mBD1-mBD3 and RT-PCR identification in MDCK cells. (**A**) Insert fragment of approximately 420 bp was separated by 2% agarose gel electrophoresis following digestion of pcDNA3.1(+)/*mBD1-mBD3* with *Eco*R I and *Xho* I. Lane1, DL15000 DNA Marker; Lane2. pcDNA3.1(+); Lane3-4, pcDNA3.1(+)/*mBD1-mBD3* digested by *Eco*R I and *Xho* I respectively; Lane5, pcDNA3.1(+)/*mBD1-mBD3* digested by *EcoR* I and *Xho* I; (**B**) The *mBD1-mBD3* mRNA expression in MDCK was analyzed by RT-PCR and the products were separated by 2% agarose gel electrophoresis. LaneM, DL700 DNA Marker; Lane1, pcDNA3.1(+); Lane2, pcDNA3.1(+)/*mBD1-mBD3*.

Recombinant plasmid pcDNA3.1(+)/*mBD1-mBD*3 was amplified by PCR and digested by *Eco*R I and *Xho* I (Figure 1A) to determine the size and orientation of the insert fragment. Insert fragment of approximately 420 bp was separated by 2% agarose gel electrophoresis, which confirmed that the size of insert fragment was consistent with *Mbd1-Mbd3* fusion gene and the correct orientation of the target insert in the eukaryotic expression vector pcDNA3.1(+). 

### 3.2. Sequence Analysis of pcDNA3.1(+)/*mBD1-mBD3*

Clones which PCR amplification and the restriction enzyme digestion showed correctly were picked and sequenced. The coding sequence of the recombinant plasmid pcDNA3.1(+)/*mBD1-mBD*3 was confirmed to be consistent with the inserted target sequence (detailed data not shown here).

### 3.3. Expression of the *mBD1-mBD3* mRNA in MDCK

Expression of *mBD1-mBD3* fusion gene in MDCK-pcDNA 3.1(+)/*mBD1-mBD3* was analyzed by RT-PCR and the products separated electrophoretically (Figure 1B). Target fragment of 420 bp was observed in MDCK-pcDNA 3.1(+)/*mBD1-mBD3* and only *â-actin* amplification fragment of 106 bp was observed in MDCK-pcDNA 3.1(+).

### 3.4. Expression of the mBD1-mBD3 Fusion Protein in MDCK

**Figure 2:**
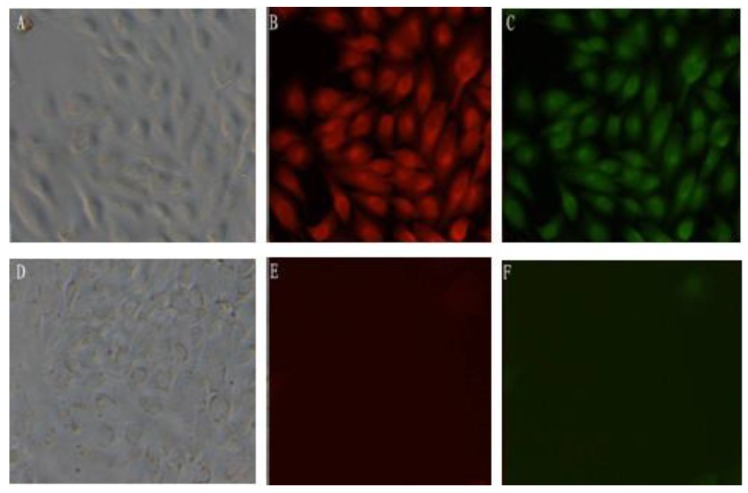
Expression of the mBD1-mBD3 fusion protein in MDCK cells (×200). Stable expression of the mBD1-mBD3 fusion protein in MDCK cells transfected with pcDNA3.1(+)/*mBD1-mBD3* or pcDNA3.1(+) was analyzed by indirect Immunofluorescence. Expressions of mBD1 and mBD3 protein emitting red and green fluorescence respectively were observed in MDCK-pcDNA 3.1(+)/*mBD1-mBD3* (**A**–**C**)*.* No fluorescence could be observed in MDCK-pcDNA 3.1(+) (**D**–**F**).

Expression of mBD1-mBD3 fusion protein in MDCK-pcDNA 3.1(+)/*mBD1-mBD3* was analyzed by indirect immunofluorescence (Figure 2). Expressions of mBD1 and mBD3 protein emitting red and green fluorescence, respectively, were observed in MDCK-pcDNA 3.1(+)/*mBD1-mBD3*. No fluorescence could be observed in MDCK-pcDNA 3.1(+).

### 3.5. Inhibition of IAV Replication in MDCK Cells

Virus titers of the cell culture supernatants of IAV-infected cells, including MDCK-pcDNA 3.1(+)/*mBD1-mBD3*, MDCK-pcDNA 3.1(+)/*mBD1*, MDCK-pcDNA 3.1(+) and MDCK) were measured using Value of TCID_50_ and −log10 of each TCID_50_ and was analyzed with SPSS v11.5 software [16] (Table 2). The TCID_50_ of pcDNA3.1(+)/*mBD1-mBD3* group was lower than other control groups significantly and the differences possessed statistical significance (*p* < 0.001). The results showed that pcDNA3.1(+)/*mBD1-mBD3* could inhibit IAV replication in MDCK cell effectively.

**Table 2:** Result of TCID_50_ titer measuring of four kinds MDCK cells (−log10, n = 3).

Groups	Supernatant virus titer (TCID_50_)
pcDNA3.1(+)/*mBD1-mBD3* group	3.4815 ± 0.1844
pcDNA3.1(+)/*mBD1* group	4.1667 ± 0.1443 *
pcDNA3.1(+) group	5.1250 ± 0.1250 **
MDCK group	5.1875 ± 0.1654 **

* *p* < 0.05 *vs*. pcDNA3.1(+)/*mBD1* group; ** *p* < 0.001 *vs*. pcDNA3.1(+) group and MDCK group.

### 3.6. Expression of the *mBD1-mBD3* Gene in Mouse Muscle

**Figure 3:**
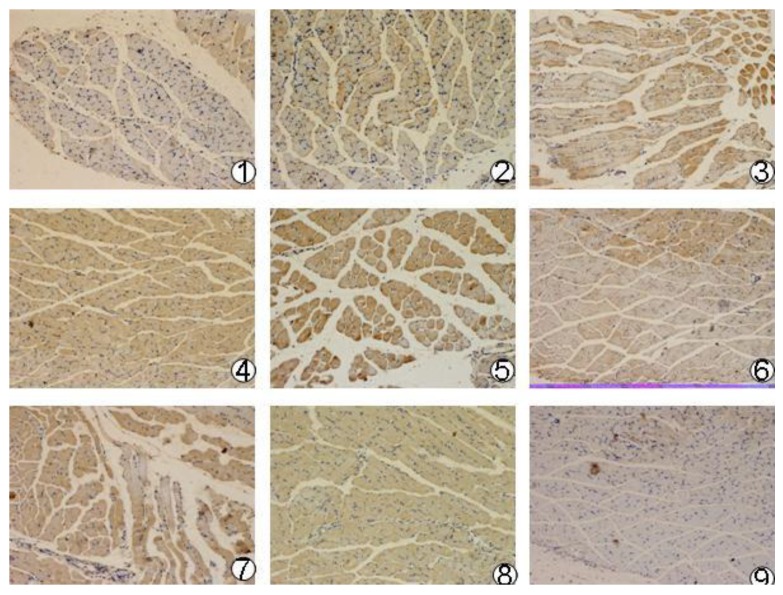
Expression of the recombinant plasmids in mouse musclar tissue (×40). Three different recombinant plasmid DNAs and pcDNA3.1(+) plasmid DNA were injected into the experimental mice and the expression of target genes were detected by immunohistochemistry after inoculation 4–72 h. There was obvious expression of recombinant protein mBD1-mBD3 showing deeper brown on cells. 1–6, pcDNA3.1(+)/*mBD1-mBD3* group at 4, 8, 16, 24, 36, 72 h after injection; 7–8, pcDNA3.1(+)/*mBD1* and pcDNA3.1(+)/*mBD3* groups at 24 h after injection, respectively; 9, pcDNA3.1(+) group at 24 h after injection.

Expression of the *mBD1-mBD3* gene in mouse muscle was analyzed by immunohistochemistry. All target proteins were detected in muscle tissue post-intramuscular injection with three recombinant plasmids (pcDNA3.1(+)/*mBD1-mBD3*, pcDNA3.1(+)/*mBD1* and pcDNA 3.1(+)/*mBD3*) during different intervals (Figure 3). There was obvious expression of recombinant protein *mBD1-mBD3* showing a deeper brown on cells at 8 h after intramuscular injection with recombinant plasmid pcDNA3.1(+)/*mBD1-mBD3*, and the expression was the best from 24 h to 36 h, until to 72 h. There was a weaker expression of recombinant protein mBD1 or mBD3 at 4 h after intramuscular injection with recombinant plasmid pcDNA3.1(+)/*mBD1* or pcDNA3.1(+)/*mBD3*, and the better expression could be detected from 12 h to 36 h, up until 72 h. Expression of the target genes were not detected in pcDNA3.1(+) negative control and PBS control during different intervals.

### 3.7. Lung Index and Lung Index Inhibitory Rates Following IAV Challenge *in Vivo*

**Figure 4:**
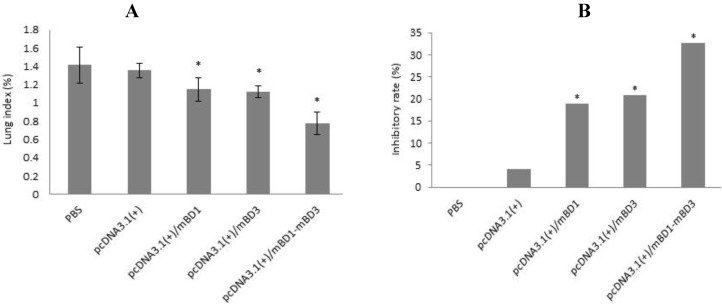
Lung index and lung index inhibitory rate. Mice were challenged with A/PR/8/34(H1N1) intranasally, and three days later three mice from each group were sacrificed and the lungs were weighed. In (**A**), a significant difference was observed in the lung index of the five groups (*p* < 0.05); In (**B**), lung inhibitory rates of 32.60%, 20.84% and 18.89% were obtained in three recombinant plasmid-treated groups, and the lung index inhibitory rate in two control groups were negligible. Data shown in Figure 4 are the mean of three samples and error bars depict the standard error.

Mice were challenged with 10× LD50 IAV intranasally, and three days later, three mice from each group were sacrificed and the lungs were weighed. The lung index and the lung inhibitory index were calculated and data were analyzed (Figure 4). The lung index of pcDNA3.1(+)/*mBD1-mBD3* group was the lowest, while those of the PBS and pcDNA3.1(+) control groups were the highest (Figure 4A). However, there was a statistical difference in the lung index between every experimental group and control group (*p* < 0.05). Lung index inhibitory rates of 32.60%, 20.84% and 18.89% were obtained in pcDNA3.1(+)/*mBD1-mBD3* group, pcDNA3.1(+)/*mBD3* group and pcDNA3.1(+)/*mBD1* group, respectively, while the lung inhibitory index in the control groups were negligible (Figure 4B).

### 3.8. Influenza Virus Titers in Lung Homogenates

Influenza virus titers in lung homogenates were tested using TCID_50_ through calculation by the Reed-Muench method and −log10 of each TCID_50_ was analyzed with SPSS v11.5 software [16] (Table 3). The TCID_50_ was 10^−6.1875^/0.1 mL, 10^−5.8750^/0.1 mL, 10^−5.250^/0.1 mL and 10^−5.1875^/0.1 mL in the PBS control group, the negative control group, pcDNA3.1(+)/*mBD3* group and pcDNA3.1(+)/*mBD1* group, respectively. There is no difference between the four groups above. However, the TCID_50_ titer was only 10^−3.4815^/0.1 mL in the pcDNA3.1(+)/*mBD1-mBD3* group. This inhibition was shown to be statistically significant (*p* < 0.005).

**Table 3:** Results of TCID_50_ titer measuring of lung homogenates (−log10, n = 3).

Groups	Lung virus titer (TCID_50_)
pcDNA3.1(+)/*mBD1-mBD3* group	3.8125 ± 0.11968 *
pcDNA3.1(+)/*mBD1* group	5.1875 ± 0.1875
pcDNA3.1(+)/*mBD3* group	5.25 ± 0.25
pcDNA3.1(+) group	5.875 ± 0.21651
PBS group	6.1875 ± 0.01875

* *p* < 0.05 *vs*. the other groups.

### 3.9. Lung Pathological Changes

**Figure 5:**
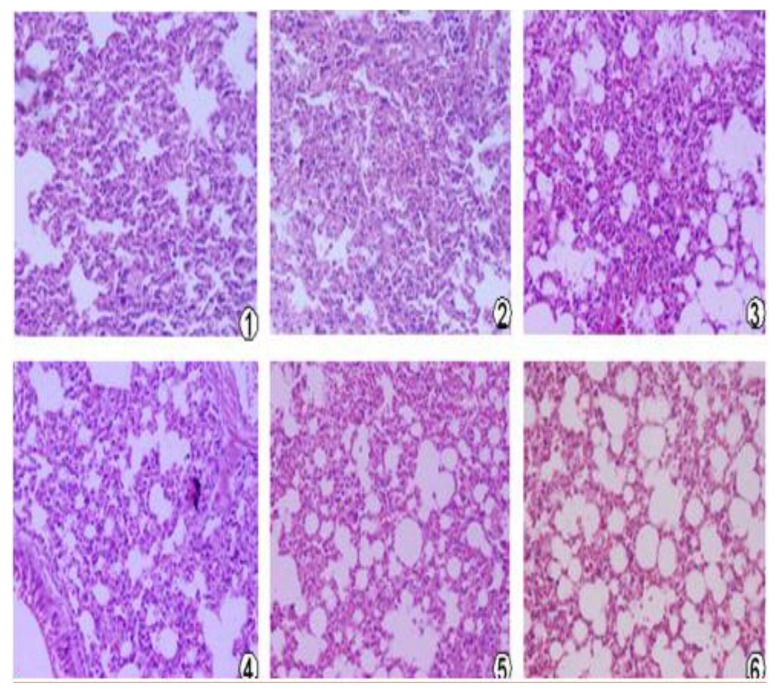
Pathological changes of the mice lung after LAV challenged (*H&E*, ×100). Mice were challenged with 10× LD50 A/PR/8/34(H1N1) intranasally, and three days later three mice of each group were sacrificed. Lung tissue was formalin-fixed, embedded in paraffin tissue blocks and sectioned for H and E staining. 1, PBS control group; 2, pcDNA3.1(+) control group; 3, pcDNA3.1(+)/*mBD1* group; 4, pcDNA3.1(+)/*mBD3* group; 5, pcDNA3.1(+)/*mBD1-mBD3* group; 6, normal control group.

Pathological changes in the lung of the mice were shown in Figure 5 (**1**–**6**). No obvious changes in the structure of the pulmonary alveolus were observed in the pcDNA3.1(+)/*mBD1-mBD3* group (**5**) and normal group (**6**). In the pcDNA3.1(+)/*mBD1*(3) and pcDNA3.1(+)/*mBD3* group (**4**) some lymphocytic infiltration were seen in the alveolar septum. This infiltration was more pronounced and accompanied by alveolar wall necrosis in the PBS control group (**1**) and the pcDNA3.1(+) negative control group (**2**).

### 3.10. IAV Challenge Survival Rate

**Figure 6:**
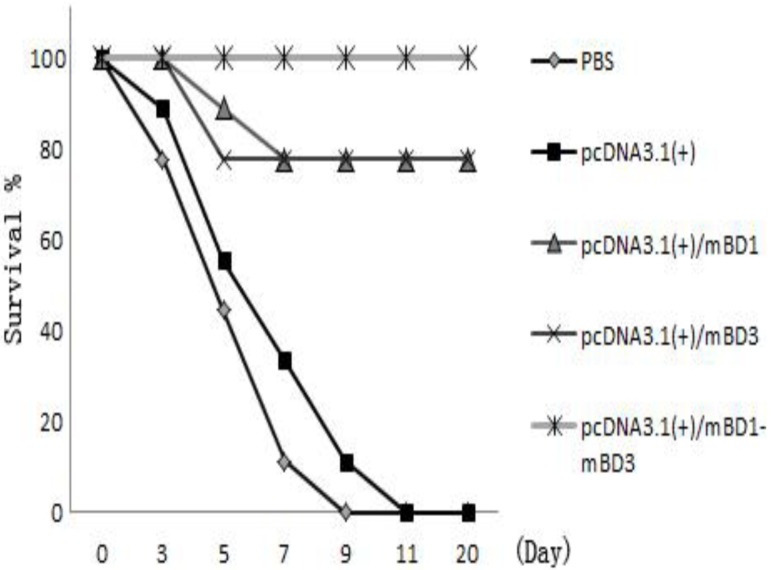
Survival rate after lethal challenge with A/PR/8/34(H1N1). The survival rates post-challenge with 10× LD50 A/PR/8/34(H1N1) are shown in Figure 6. No mice survived from the control groups, seven mice survived in pcDNA3.1(+)/*mBD1* group and pcDNA3.1(+)/*mBD3* group, and all the mice survived in pcDNA3.1(+)/*mBD1-mBD3* group. Significant differences in the survival rates of the three recombinant plasmid-treated groups were observed relative to the control groups (*p* < 0.05).

The survival rates post-challenge are shown in Figure 6. Survival rates of mice challenged with a lethal dose of IAV showed that administration of pcDNA3.1(+)/*mBD1-mBD3* is protective in this model. No mice survived in the two control groups. Seven mice survived in the pcDNA3.1(+)/*mBD1* group or pcDNA3.1(+)/*mBD3* group, while the survival rates of the two groups all reached 77.78%. No single mouse died in pcDNA3.1(+)/*mBD1-mBD3* group and the survival rates reached 100%. The survival rates in the three groups administered recombination plasmids were significantly different relative to the control groups (*p* < 0.05).

## 4. Discussion

At present, the influenza vaccines used mainly utilize inactivated influenza virus or recombinant viral glycoprotein to induce immune response by intramuscular injection. This immune protection based on IgG antibody would become weaker gradually within 6 months. For those susceptible, such as the aged, or people with impaired immune systems, the effective rate of the influenza vaccines only reaches 39%. Furthermore, other reasons of vaccine utility being limited include the lack of timely manufacturing of sufficient vaccine and the potential for antigenic mismatches with circulating strains [17,18]. Although some anti-virus drugs that have been approved on the market could be used to prevent and treat influenza, their applications are limited for severe side effects and drug resistance. As a result, some effective agents on the prevention and treatment of influenza have become the pressing issue.

Natural antiviral defenses are mediated by the innate and adaptive immune systems. Adaptive immune responses to one strain of IAV do not afford lasting protection such as epidemic IAV. Innate immune mechanisms appear to provide some way of protection against IAV. Recently, several defensins were shown to inhibit IAV via avariety of mechanisms *in vitro*. For example, the synthetic primate θ-defensin retrocyclin 2 (RC2) and human β-defensin 3 (HBD3) have been showed to block viral infection by directly related to their ability to cross-link cell membrane glycoproteins into a fusion-resisting barricade [19], whereas α-defensins human neutrophil peptides (HNPs) induce impairment of cellular pathways and increase neutrophil activity [20]. To be the important composition of innate immunity, β-defensin can kill pathogen by rapidly releasing and directly attacking when tissues are damaged for infection. It can also enhance the activity of dendritic cell (DC) of absorbing, processing and disposing antigen through mediation of chemotactic factor-receptor 6 (CCR6), sequentially presenting antigen to the initial T cell, activating T cell and initiating acquired immunity [21,22]. Thus, defensin is a link between innate immunity and acquired immunity. Mouse β-defensins (mBD) possess higher homology with the human β-defensins, and the *mBD1* gene can constitutively express and the *mBD3* gene can induce expression in mouse. Moser and Grubor *et al*. reported that mBD can reduce mRNA level of parainfluenza virus (PIV) in mouse lung tissue and put off infection of PIV effectively [23,24]. Although neither mBD1 or mBD3 possess antivirus activity, their antivirus activity is weaker when they are used individually. A study shows that mBD 1 and mBD 3 can kill effectively enveloped viruses such as human immunodeficiency virus (HIV) or vesicular stomatitis virus (VSV), but they are useless for naked viruses without envelopes such as cytomegalovirus (CMV), reovirus and echovirus [25,26,27]. 

Because of its sequencing convenience, well growing in selective medium containing G_418_ and expressing character of exogenous DNA, eukaryon expression plasmid pcDNA3.1(+) containing T7 promoter and *neo* gene is commonly used as vector to express exogenous peptides. In this study, the employed expression vector is plasmid pcDNA3.1(+). Firstly, we connected *mBD1* gene and *mBD3* gene through a link peptide of five amino acids by overlap-PCR, and constructed a pcDNA3.1(+)/*mBD1-mBD3* recombinant plasmid successfully. Secondly, we obtained MDCK cells with a stable content pcDNA3.1(+)/*mBD1-mBD3* through screening of G_418_. RT-PCR confirmed that *mBD1-mBD3* mRNA can continue expression in this MDCK cell line, and immunohistochemistry analysis showed that mBD1-mBD3 fusion protein expressed mainly in cytoplasm and nucleus. Virus production in culture supernatant of MDCK-pcDNA 3.1(+)/*mBD1-mBD3* were inhibited more than 50-fold relative to the control and were only inhibited four-fold relative to MDCK-pcDNA 3.1(+)/*mBD1.* This result was found to be reproducible on two further occasions. These obtained results showed that pcDNA3.1(+)/*mBD1-mBD3* can effectively inhibit influenza virus PR8 production in the culture supernatant and the fusion expression of *mBD1* gene and *mBD3* gene can enhance this inhibitory effect.

Then, we observed the expression of pcDNA3.1(+)/*mBD1-mBD3* recombinant plasmid *in vivo*. We found there was obvious expression of the recombinant protein mBD1-mBD3 showing a deeper brown stripe on the cell membrane or a deeper brown spot in the cytoplasm at 8 h after intramuscular injection with liposome-encapsulated pcDNA3.1(+)/*mBD1-mBD3* through immunohistochemistry, and the expression was the best from 24 h to 36 h, until 72 h. But expression of the target genes were not detected in pcDNA3.1(+) negative control and PBS control during different intervals. The results have shown that pcDNA3.1(+)/*mBD1-mBD3* recombinant plasmid can express in mice myocyte successfully. Based on above results, mice were challenged with a lethal dose of influenza virus after eight hours post-inoculation with pcDNA3.1(+)/*mBD1-mBD3*. The structure of the pulmonary alveolus remained unchanged in the pcDNA3.1(+)/*mBD1-mBD3* group, while lymphocytic infiltration and tissue necrosis was observed to varying degrees in the alveolar septum of mice in the other groups. Analysis of lung index and lung inhibitory index also showed that there was least inflammation in the lungs of the pcDNA3.1(+)/*mBD1-mBD3* group. Detection of TCID_50_ also showed that influenza virus production was effectively inhibited in the pcDNA3.1(+)/*mBD1-mBD3* group and the protective rate of mBD1-mBD3 with mice against influenza virus can reach to 100%. We have adopted intramuscular injection to deliver eukaryotic expression vector in this study, and all the results have shown that the recombinant mBD1/mBD3 expressed in the muscle manifest anti-influenza in the lung indeed. Based on the results, we guessed that the recombinant mBD1/mBD3 may be transported to the other tissues (such as lung, spleen or liver *etc*.) through body fluid, then play anti-virus activity in body other tissues.

In the present study, we demonstrated that the pcDNA3.1(+)/*mBD1-mBD3* recombinant plasmid can express mBD-mBD3 fusion protein successfully *in vitro* and *in vivo*. Furthermore, we have verified the effect against influenza virus of the mBD-mBD3 recombinant protein *in vitro* and *in vivo*. This data represents an experiment foundation for further research into the molecular mechanism of anti-influenza virus with defensin. Although we also found that there may be some important interaction between mBD1 gene and mBD3 gene, such as overlapping regulatory roles, the specific mechanism of their interaction should be explored further in the future.

## 5. Conclusions

The mBD1-mBD3 could be expressed by the recombinant plasmid pcDNA3.1(+)/*mBD1-mBD3*, and inhibit influenza A virus replication both *in vitro* and *in vivo* effectively. This recombinant mBD1-mBD3 might be developed into an agent for influenza prevention and treatment.
